# Staff perceptions of addressing lifestyle in primary health care: a qualitative evaluation 2 years after the introduction of a lifestyle intervention tool

**DOI:** 10.1186/1471-2296-13-99

**Published:** 2012-10-10

**Authors:** Siw Carlfjord, Malou Lindberg, Agneta Andersson

**Affiliations:** 1 Department of Medical and Health Sciences, Division of Community Medicine, Linköping University, Linköping, SE, 581 83, Sweden; 2 R&D Department of Local Health Care, County Council of Östergötland, Linköping University, Linköping, Sweden; 3 Department of Medical and Health Sciences, Division of Primary Care, Linköping University, Linköping, Sweden

**Keywords:** Staff perception, Lifestyle counselling, Primary health care, Computerized tool

## Abstract

**Background:**

Preventive services and health promotion in terms of lifestyle counselling provided through primary health care (PHC) has the potential to reduce morbidity and mortality in the population. Health professionals in general are positive about and willing to develop a health-promoting and/or preventive role. A number of obstacles hindering PHC staff from addressing lifestyle issues have been identified, and one facilitator is the use of modern technology. When a computer-based tool for lifestyle intervention (CLT) was introduced at a number of PHC units in Sweden, this provided an opportunity to study staff perspectives on the subject. The aim of this study was to explore PHC staff’s perceptions of handling lifestyle issues, including the consultation situation as well as the perceived usefulness of the CLT.

**Methods:**

A qualitative study was conducted after the CLT had been in operation for 2 years. Six focus group interviews, one at each participating unit, including a total of 30 staff members with different professions participated. The interviews were designed to capture perceptions of addressing lifestyle issues, and of using the CLT. Interview data were analysed using manifest content analysis.

**Results:**

Two main themes emerged from the interviews: a challenging task and confidence in handling lifestyle issues. The first theme covered the categories responsibilities and emotions, and the second theme covered the categories first contact, existing tools, and role of the CLT. Staff at the units showed commitment to health promotion/prevention, and saw that patients, caregivers, managers and politicians all have responsibilities regarding the issue. They expressed confidence in handling lifestyle-related conditions, but to a lesser extent had routines for general screening of lifestyle habits, and found addressing alcohol the most problematic issue. The CLT, intended to facilitate screening, was viewed as a complement, but was not considered an important tool for health promotion/prevention.

**Conclusion:**

Additional resources, for example in terms of manpower, may help to build the structures necessary for the health promotion/prevention task. Committed leaders could enhance the engagement among staff. Cooperation in multi-professional teams seems to be important, and methods or tools perceived by staff as compatible have a potential to be successfully implemented. Economic incentives rewarding quantity rather than quality appear to be frustrating to PHC staff.

## Background

Preventive services and health promotion in terms of lifestyle counselling provided by primary health care (PHC) has the potential to reduce morbidity and mortality in a number of areas, such as coronary heart disease and diabetes [1-3]. In Sweden, the Public Health Policy adopted by the government in 2003 states that a more health-promoting and disease-preventative perspective should permeate all health services and be an obvious part of all care and treatment [4]. National guidelines on methods for preventing disease, published in 2011, recommend that individuals known to have a lifestyle that is potentially harmful to health should be given lifestyle counselling [5]. As one of many actors in the community, PHC has a responsibility for public health, and has an obligation to provide preventive services to the population [6]. However, due to lack of time, resources and skills, health promotion and preventive services are not provided to a degree that corresponds to the needs of the population [7-12]. In a qualitative study performed in Swedish PHC, Johansson et al. [13] identified existing values, structures and resources as barriers to providing health promotion/preventive services. Health professionals in general were positive about and willing to develop a health-promoting and/or preventive role, but they called for organizational changes and more explicit leadership [13]. District nurses in Sweden in general find health promotion/prevention important, but state that medical matters are given priority over activities to promote health and prevent disease [14]. Other obstacles to health promotion/prevention in daily practice identified by former research are lack of guidelines and unclear objectives [15].

Modern technology can be used to overcome some of the barriers perceived by PHC staff, and computer-delivered interventions have been evaluated and found effective in various settings [16,17]. To be effective, however, such tools have to be implemented and integrated into routine practice [18]. In 2008, a computer-based tool for lifestyle intervention (CLT) was introduced at a number of PHC units in Sweden, with the intention of facilitating the delivery of health-promoting and preventive services [19]. To study staff perspectives on the implementation, a qualitative evaluation was performed after 9 months, showing that staff expectations, perceived compatibility and perceived advantage were important factors influencing how the tool had been used [20]. After 2 years a second evaluation was performed, of which the present study forms part.

As already described, research regarding how to address lifestyle issues in Swedish PHC has mainly focused on barriers and to some extent possibilities or facilitators [13-15]; staff perceptions about available resources and tools, or how these are used, have not been assessed to the same degree. The present study was performed with the aim of exploring PHC staff’s perceptions of handling lifestyle issues, including the consultation situation as well as the perceived usefulness of a lifestyle intervention tool.

## Methods

A qualitative design with focus group interviews was chosen for the study. The study population consisted of staff from six PHC units (i.e. health care centres with general practitioners (GPs) and other health care professionals), where the CLT was introduced in 2008 [19]. Before the introduction of the CLT, an invitation was directed to the public health administration of three county councils in southeast Sweden, and six PHC units, two from each of the counties, were asked to participate. The CLT includes assessment and tailored advice regarding alcohol consumption and physical activity, and is described in detail by Carlfjord et al. [21]. At the introduction of the CLT, staff members were asked to encourage their patients to perform the lifestyle assessment. In the 2 years since the introduction, staff have received regular written feedback based on data registered in the CLT database.

### Data collection

After the CLT had been in operation for 24 months, staff members at each of the six units were invited to participate in a focus group interview. A message was sent to the manager at each unit, with a request to invite 1–2 individuals from each staff category (GPs, nurses, nurse assistants (NAs) and, when available, allied professionals (APs)) for the interviews. These staff members had direct contact with patients, and thus could be expected to have had the opportunity to refer patients to the CLT. Those who signed up for the interviews and showed up at the interview session, a convenience sample, were included as informants. Six focus group interviews were conducted, one at each participating PHC unit, involving 30 staff members (nine GPs, 12 nurses, six NAs, three APs). All participants, except for one nurse and four GPs, were women. Focus group size varied from four to six members; the average was five. The interviews were performed during the lunch hour, lasted between 34 and 43 min (mean 40 min) and were conducted in a room used for staff meetings on the premises of each PHC unit. The author (S.C.) served as moderator and the interviews were observed by an assistant who took notes. As recommended by Krueger, the moderator and the assistant had a brief talk about their impressions after each session [22]. The interviews took place between April and December 2010.

Two scenarios were prepared for discussion among the informants. First, a patient case was presented by the moderator (S.C.), showing a middle-aged woman and a description of symptoms such as headache and sleep disorders. The informants were asked one general question on how they would handle the patient. The purpose was to trigger a discussion on how the informants act in a consultation with such a patient. The second scenario, read by the moderator (S.C.), described a PHC unit in another part of the country that had been offered to try the CLT and was now asking for advice – what do you tell them? The goal was to start a discussion on how the informants perceived the role of the CLT in health promotion/prevention. Interviews were recorded using a digital recorder and were transcribed verbatim by an assistant. After transcription, the moderator (S.C.) listened to the recordings and read all the transcribed material, and made corrections if necessary.

### Data analysis

Interview data were analysed using the manifest content analysis method according to Graneheim and Lundman [23]. The unit of analysis was the transcribed interviews. The text was first read and re-read by the authors of this article. An inductive approach was used and meaning units, i.e. words and sentences containing aspects related to each other, were extracted from the text and labelled with codes. The coding was performed by the first author (S.C.), and was discussed and negotiated in a peer debriefing session with the other authors (M.L., A.A.), as described by Polit and Beck [24]. Codes with similar content were then sorted into subcategories, subcategories were sorted into categories and two main themes emerged. Main themes are, according to Graneheim and Lundman [23], themes that express the underlying meaning of the manifest content throughout categories and subcategories. Thus codes, subcategories, categories and themes all emerged from the interviews. The process of categorizing and identifying themes was discussed until consensus was reached to enhance the trustworthiness of the findings. A computer-based system, NVivo 9, was used as an aid to sort and analyse the interview data.

### Ethics

Respondents volunteered to participate in the study. The respondents were informed that all data collected would be treated confidentially, and the results presented so that no individuals could be identified. The study was approved by the Ethics Board in Linköping, Sweden (Dnr Ö 16–08).

## Results

Data analysis resulted in two main themes regarding addressing lifestyle issues in PHC: a challenging task and confidence in handling lifestyle issues. The first theme covers the categories responsibilities and emotions, and the second theme covers the categories first contact, existing tools, and role of the CLT. In the quotations, the authors’ explanations appear in brackets, … means hesitation and (…) means that some words have been left out.

### Challenging task

#### Responsibilities

Regarding responsibilities, three subcategories emerged from the interviews: patient responsibilities, care giver responsibilities and responsibilities at the political level.

The respondents thought that the patient’s autonomy is very important and must be respected. Preventive services should be offered, but the decision about whether to accept them rests with the patient. A prerequisite for effective health promotion/prevention is a motivated patient, a motivation that, however, might be facilitated by the care giver.

"(…) well, they have to reach that point themselves that they understand that no one else can take responsibility for their lives, the health care system shouldn’t do that, or can’t do that (giggle), they have to do it on their own (…) (Nurse Unit I)"

"(…) but sometimes the motivation can come after you’ve talked it over … so if they just make it to the appointment that’s good (NA Unit II)"

Many patients were perceived as having false expectations regarding PHC, and believe that there is always a quick fix. For example, patients ask for drugs to make them feel better allowing them to go back to work as soon as possible, because they do not have time to be absent. The informants expressed that patients often deny problems related to lifestyle, do not see how they themselves can contribute to their own health, and at the same time do not allow themselves to be ill.

"(People) just don’t have time for anything today, to fix on their own, she just wants pills so she can go straight back to work (Nurse Unit I)"

The staff members were also concerned about the responsibilities they, the caregivers, have when providing preventive services in terms of advice regarding lifestyle. If patients were referred to the CLT, the staff found it very important that some kind of follow-up was offered. It was also mentioned that the CLT is not enough to provide health promotion/prevention; other activities, like for example individual counselling, are needed as well.

"It’s not enough to set up one of these (the CLT) at the health care centre and think that you fill the criteria (for preventive services) (GP Unit II)"

The respondents thought that additional resources were essential if preventive services are to be provided to the population in an effective way. Among the PHC units participating in the study, some had received additional resources in terms of manpower, others had not. Those who had resources were very satisfied with this and seemed to handle lifestyle issues in a more structured way and with more confidence. Those who lacked resources described how they struggle to make the best of the existing situation.

"(…) sure, it would have been great if we’d had extra resources, but we don’t have any extra resources, so we are working small miracles, and this is how we do it (GP Unit III)"

Regarding priorities and political goals, the staff members stated that when resources are scarce and acute situations come up, like for example immunization campaigns, addressing lifestyle behaviours receives less priority. They also expressed disappointment with politicians formulating goals that are perceived as impossible to reach in practice.

"We’ve had to cut down on preventive work, you don’t prioritize that when there’s not enough time for everything you want to do (GP Unit IV)"

"Politicians set up goals that we can’t live up to (GP Unit V)"

Another issue mentioned by the informants was financial incentives. Some staff members expressed positive opinions regarding financial incentives on a unit level. Others were negative, arguing that financial incentives often give money for activities that are easy to measure, encouraging quantity rather than quality.

"(…) and then you can take availability for instance, or how many prescriptions on physical activity that we make and things like that, but if the patients get well in the end, no one asks about that, we don’t get any money for that (…) (GP Unit V)"

#### Emotions

The emotions category concerns feelings of commitment to addressing lifestyle issues and perceived difficulties. Among PHC staff, there is a certain commitment to addressing lifestyle issues. In general they believe that it is an important part of their work, and they describe how a lifestyle clinic, team or nurse is a valuable resource. However, at one of the units, there was a certain air of fatigue regarding the issue, which was explained by the fact that enthusiasts for health promotion/prevention no longer worked at the PHC.

"It was [Name] and [Name] who were involved in that lifestyle project, they both quit … the two real enthusiasts (Nurse Unit IV)"

Leadership was also mentioned, and staff emphasized the importance of a manager committed to health promotion/prevention both with regard to addressing lifestyle issues in general, and to the implementation of a new tool such as the CLT.

"(…) that the manager thinks that this is a good and important thing that we are supposed to work with, so that it’s well supported and has a natural place (Nurse Unit V)"

Despite the interest in economic incentives, there were also voices showing a deeper understanding of the positive outcomes linked to addressing lifestyle. One person described how she perceived the possibility of providing adequate help to a patient in need as a reward.

"(…) actually, my reward isn’t that we get extra money or anything but that I have proper help to give to those who need it (GP Unit II)"

A more common experience was that lifestyle issues tend to be forgotten when the work load is increasing, and despite interest in the subject and awareness of the importance, less is done than what is intended. A difficulty perceived by a number of staff members was to initiate a conversation about lifestyle, particularly alcohol consumption, when the patient is seeking care for something else.

"(…) at other times we’re actually not, we don’t really … we just don’t focus on that [lifestyle issues] (AP Unit V)"

At one unit, however, staff expressed a feeling of resignation looking back at the situation a couple of years ago when a manager committed to working with lifestyle issues and other enthusiastic staff members were present at the PHC.

"We did have a true enthusiast here, our last manager, maybe that’s what we need again (…) he made us do what we didn’t even have time for (GP Unit IV)"

Alcohol consumption was the area for which most informants perceived difficulties in asking questions and providing advice. It became easier if included in a package with other lifestyle areas, and at some units this was solved by using a lifestyle assessment form.

"From my point of view, when I’m on the phone, I find it sort of difficult to ask about drinking habits… it’s … we have a slip of paper [a questionnaire] that we use, it gives you something to hold on to, but sometimes we don’t have that much time for each call either … and it feels sort of embarrassing really, I must say that I’m just not very good at it (Nurse Unit IV)"

### Confidence in handling lifestyle issues

#### First contact

The respondents seemed to be confident about how to handle a patient seeking help for a condition that could be related to lifestyle habits. However, they stressed the importance of thoroughly assessing the history of the patient and making a somatic examination to first rule out somatic illness. In general, they also showed confidence in how to ask about lifestyle habits potentially harmful to health, respecting patient autonomy and encouraging patient initiatives to change. Nurses seemed to know when to consult a GP, and GPs had confidence in the competence of the nurses, NAs and APs, and referred to these groups for interventions.

"(…) at first you need to ask if she thinks that there may be an underlying cause and then I guess there must be a somatic examination and some tests … just to rule out physical illness (…) (GP Unit IV)"

"(…) and then I check back with the doctor, of course, otherwise I refer the patient to an addiction rehab clinic if that seems necessary, I never keep a patient I always refer them, I try to give advice and find solutions (AP Unit III)"

A common measure taken is to provide advice, not just once but also offering follow-up. Recommending physical activity was mentioned in many of the groups as a first step. The CLT, however, was not considered suitable as a first choice when a patient with a lifestyle-related condition contacts the PHC unit.

"(…) so in that first stage I would never have recommended the CLT, I don’t think so (Nurse Unit I)"

If the patient was considered to need more than just advice and follow-up, staff mentioned a number of possibilities for referral, described in further detail below.

#### Available tools

Staff members at most of the units expressed satisfaction with the way lifestyle issues are currently handled. At some of the units, addressing lifestyle issues was organized as a lifestyle clinic, a lifestyle team or a lifestyle nurse, trained to handle those patients after referral from other staff members. Respondents also mentioned the possibility of referral to a physiotherapist, dietician, counsellor, or prescribing physical activity. At most of the units, staff members expressed preparedness for this type of patient. However, at some units, it was obvious that patients with a severe condition were the only ones that could be handled due to scarce resources, and that those with less complexity could not be offered continuous support.

"(…) and then we have a health coach nurse that you can refer patients to and who goes through these, well, the health profiles that you do (Nurse Unit VI)"

Confidence in handling a patient with a lifestyle-related condition, and satisfaction with the organization, was particularly expressed by informants who had access to a lifestyle clinic, a lifestyle team or a lifestyle nurse within the unit.

"An advantage is that we have this structure, and that everyone knows how it all works (Nurse Unit II)"

"(…) and then they get a questionnaire including all the health-related areas, food and exercise and sleep and alcohol and smoking and all of that, and then they also get this brochure that we have, with some advice and tests … all in the same letter, and after that I give them a call after one or two weeks to follow up and ask if they have filled out the form or if they have any questions (…) (AP Unit III)"

The CLT was mentioned as a possibility for patients who choose to perform the lifestyle assessment without actually being referred, or as a complement, despite its limitations.

"(…) and also the computer [the CLT] of course, even if it doesn’t cover everything, you only have questions about exercise and alcohol there, so to speak, but I could recommend something like that as a professional (NA Unit V)"

An assessment form that was filled in by the patient or by the caregiver also was mentioned as a simple but very useful tool. It seems that this helped the caregiver to address the issue in an easy way.

"If I’ve been fortunate, the nurse has already sent out a questionnaire that covers the four main areas that I ask about, exercise, food, tobacco and alcohol, so this is a very good place to start (GP Unit IV)"

#### Role of the CLT

Where work with lifestyle issues was already organized and functioning well, the CLT was perceived unnecessary, and was not thought to add anything new. Possible advantages mentioned were that the lifestyle assessment is performed anonymously, and that the CLT could be a complement to other activities. When the CLT is introduced in a new setting, staff members expressed the importance of involvement in the decision to use it and the possibilities to influence its use. There must be a commitment to using the CLT, and it should be part of a larger package on how to deal with lifestyle issues.

"That everyone gets the same information, and that it’s presented at a staff meeting to make you feel that you’ve been a part of it all, that you’ve had the possibility to have a say so before it’s all launched (Nurse Unit V)"

"It should be a part of how you work, (…) when you have a patient like this, if I think it’s the right thing (…) I can turn the patient over to a nurse who uses the computer [the CLT] as a tool … I can see that as an option (…) (GP Unit V)"

## Discussion

Many of the PHC staff members participating in the study expressed commitment to working with lifestyle issues and find it challenging. Work with health promotion/prevention is organized in a way perceived by staff members as satisfying at most of the units, and staff expressed confidence in how to handle patients presenting with lifestyle-related conditions. Initiating a discussion about lifestyle with a patient attending the centre with a non-lifestyle-related condition is perceived as problematic. Additional resources in terms of manpower seem to increase the possibilities of handling the issue in a structured way.

The CLT, introduced to facilitate a more general screening and identify potentially harmful lifestyle behaviour, is not seen as an essential part in health promotion/prevention in PHC 2 years after its introduction. When evaluated after 9 months, compatibility with existing routines was one of the factors associated with a successful implementation [20]. It is possible that perceived lack of compatibility resulted in little interest in incorporating the CLT into routine practice despite a commitment to addressing lifestyle issues. At some of the units other tools for lifestyle screening are used and found feasible.

Patients, caregivers, managers and politicians are considered to have responsibilities regarding how lifestyle issues are handled. Among staff, there is a disappointment regarding unrealistic goals set by politicians, and with evaluations giving higher value to quantity than quality in care.

In earlier research, a number of barriers to addressing lifestyle issues in PHC were identified [7,14,15], and health care staff have expressed how structures, values and lack of resources are limiting their chances to promote health [13]. Possibilities or facilitators identified include commitment to work with health promotion/prevention, knowledge, resources and attitudes [14,15]. The present study shows how these possibilities, at most of the PHC units included in the study, have been converted into health promotion/prevention activities perceived by staff as satisfying. Awareness about lifestyle issues might have been influenced by participation in the study, but most of the commitment and activities described are not connected to the CLT, which is merely seen as a complement or as one tool among others used to address lifestyle issues. Other such tools have been evaluated and found feasible, e.g. a single checklist reminder or the Health Square [25,26]. It seems that, when staff at the local unit find tools that fit their organization and are perceived as compatible with existing routines, they incorporate them in their practice [27].

When the staff in the present study discussed responsibilities, they clearly stated that patients, staff and politicians all play important roles. They saw themselves as providers of knowledge and guidance to patients with respect for patient autonomy, and they also try to fulfil expectations from stakeholders; however, the latter is not always considered possible. Demands in health care organizations are continuously increasing, and it takes strong leaders to maintain engagement among staff under those circumstances [28]. Staff in the present study called for manager commitment, showing a demand for strong leadership. Another important issue mentioned in the interviews was that stakeholders do not always value quality in care, but use quantitative measures as a basis for economic incentives. Among the staff, quality is perceived more important than quantity, and to provide adequate help to a patient in need is seen as a reward.

In earlier studies, PHC staff expressed that additional resources for lifestyle intervention are needed but not always provided [12,13]. In the present study, some units had received additional resources, others had not. The general impression was that, where resources were provided, these had been utilized as intended, and staff felt content with the situation. Among those who had not received additional resources, staff seemed to use available resources in a judicious way. However, if so called lifestyle clinics are to be put into practice additional resources seem to be crucial.

Staff members in PHC, according to this study, feel well prepared to handle patients presenting with lifestyle-related conditions. PHC staff also seemed confident about their respective professional and individual roles; they know when to act on their own and when to refer to a colleague. This kind of team-based practice has been found to improve patients’ perceptions of quality of care and confidence in the system [29]. However, general screening for lifestyle issues among patients seemed not to be prioritized.

Addressing lifestyle issues seemed to be more complicated at one of the units than the others. At this particular unit, there had been a flourishing activity until a couple of years ago, when two staff members, enthusiastic and committed to health promotion/prevention, quit their employment. Program champions have been found to be very important for organizational change [30], but results from the present study shows the vulnerability associated with building activities on a few enthusiastic individuals.

The study was performed using a qualitative method in accordance with the RATS guidelines [31]. Some limitations need to be taken into account in the interpretation of the results. Results from qualitative research are not generalizable to other settings, and to enable the reader to evaluate whether the results could be applied in a similar context, the setting and the participants are described as thoroughly as possible without revealing their identity. Quotations from the interviews are provided throughout the results section to obtain credibility and authenticity. During the analysis, the authors repeatedly discussed the interpretation of findings to ensure criticality and integrity. These measures are taken to develop trustworthiness of a qualitative study as suggested by Guba and Lincoln [32]. Most of the quotes represent GPs and nurses, which only partly reflects the composition of the focus groups; NAs and APs constituted almost a third of the participants. GPs and nurses, however, were those who contributed most to the discussion; NAs did not participate as actively as the others. Performing the interviews in mixed groups might have limited the contribution of data from some of the participants due to hierarchic structures among staff. However, the authors believed that it was important that the different professionals could interact and describe their collaboration, and thus mixed groups were considered the most appropriate method for the study. Another limitation is the use of a convenience sampling method, which may have limited the representativeness of the informants. The results could also have been influenced by the fact that the researcher moderating the interviews was the same person who introduced the CLT [19]. A suggestion for further research in this area is to assess managers’ views on how lifestyle-related conditions should be addressed in PHC, and also to compare the opinions of the different professional groups.

The results of the study show that PHC staff perceive addressing lifestyle issues as important and challenging, and they express confidence in handling patients with lifestyle-related conditions. The most problematic issue for the staff is addressing alcohol, and they call for a structured method of handling this issue.

## Conclusion

To conclude, the study results suggest that additional resources, for example in terms of manpower, may help to build the structures necessary for the task. Committed leaders could enhance the engagement among staff. Cooperation in multi-professional teams seems to be important, and methods or tools perceived by staff as compatible have a potential to be successfully implemented. Economic incentives rewarding quantity rather than quality appear to be frustrating to PHC staff.

## Competing interests

The authors declare that they have no competing interests.

## Authors’contributions

SC participated in the design of the study, the data collection, the analysis of data and drafted the manuscript, ML participated in the design of the study, the analysis of data and helped to draft the manuscript, AA participated in the design of the study, the analysis of data and helped to draft the manuscript. All authors read and approved the final manuscript.

## Pre-publication history

The pre-publication history for this paper can be accessed here:

http://www.biomedcentral.com/1471-2296/13/99/prepub
